# Obstetrician involvement in planned midwife-led births: a cohort study in an obstetric department of a University Hospital in Switzerland

**DOI:** 10.1186/s12884-021-04209-2

**Published:** 2021-10-27

**Authors:** Ann-Katrin Morr, Nicole Malah, Andrea Manuela Messer, Annina Etter, Martin Mueller, Luigi Raio, Daniel Surbek

**Affiliations:** Department of Obstetrics and Gynecology, University Hospital Inselspital Bern, University of Bern, 3010 Bern, Switzerland

**Keywords:** Midwife-led birth care, Secondary obstetrician involvement, Birth modes, Maternal and neonatal outcome

## Abstract

**Background:**

Healthy women with low risk singleton pregnancies are offered a midwife-led birth model at our department. Exclusion criteria for midwife-led births include a range of abnormalities in medical history and during the course of pregnancy. In case of complications before, during or after labor and birth, an obstetrician is involved. The purpose of this study was 1) to evaluate the frequency of and reasons for secondary obstetrician involvement in planned midwife-led births and 2) to assess the maternal and neonatal outcome.

**Methods:**

We analyzed a cohort of planned midwife-led births during a 14 years period (2006-2019). Evaluation included a comparison between midwife-led births with or without secondary obstetrician involvement, regarding maternal characteristics, birth mode, and maternal and neonatal outcome. Statistical analysis was performed by unpaired t-tests and Chi-square tests.

**Results:**

In total, there were 532 intended midwife-led births between 2006 and 2019 (2.6% of all births during this time-period at the department). Among these, 302 (57%) women had spontaneous vaginal births as midwife-led births. In the remaining 230 (43%) births, obstetricians were involved: 62% of women with obstetrician involvement had spontaneous vaginal births, 25% instrumental vaginal births and 13% caesarean sections. Overall, the caesarean section rate was 5.6% in the whole cohort of women with intended midwife-led births. Reasons for obstetrician involvement primarily included necessity for labor induction, abnormal fetal heart rate monitoring, thick meconium-stained amniotic fluid, prolonged first or second stage of labor, desire for epidural analgesia, obstetrical anal sphincter injuries, retention of placenta and postpartum hemorrhage. There was a significantly higher rate of primiparous women in the group with obstetrician involvement. Arterial umbilical cord pH < 7.10 occurred significantly more often in the group with obstetrician involvement, while 5′ Apgar score < 7 did not differ significantly. The overall transfer rate of newborns to neonatal intensive care unit was low (1.3%).

**Conclusion:**

A midwife-led birth in our setting is a safe alternative to a primarily obstetrician-led birth, provided that selection criteria are being followed and prompt obstetrician involvement is available in case of abnormal course of labor and birth or postpartum complications.

## Background

In Switzerland, as in many high-income countries, care during labor and birth is mostly performed by obstetricians and midwives, and birth mainly takes place in hospital-based obstetric units with obstetrician-midwife-team settings of birth.

In 2019 there were a total of 86,172 births in Switzerland [1]. According to current statistics, women in Switzerland give birth in a hospital-based obstetric unit in 97%, mostly in a primarily obstetrician-led birth setting [2]. Roughly two thirds of all births (68%) are vaginal births (among these one sixth instrumental vaginal births), and about 32% are caesarean sections [2]. While caesarean section rate has risen over the last 2 decades in Switzerland, it has reached a plateau in the last 4 years. Only about 3% of births in Switzerland take place outside a hospital: in birth centers, at home or abroad [2]. These numbers are comparable to other European countries (e.g. Germany).

Some specific countries in the EU (e.g. the U.K. and the Netherlands) have a traditionally higher rate of women giving birth at home or in midwife-led birth settings. In Switzerland midwife-led births mainly take place outside of hospitals (birth centers or at home) and only a few midwife-led birth models are being offered in clinical settings of obstetric departments. According to the statistic report of the Swiss Midwife Association, there were 5241 midwife-led births in 2019: 38.0% in birth centers, 16.9% at home, 32.3% performed by a free practicing midwife in a hospital. 12.3% women had to be transferred to a hospital or needed secondary obstetrician involvement during labor led by free practicing midwives within the hospital due to abnormalities, complications or desire for epidural anaesthesia. For 0.5% women no information is available [3].

Throughout the last decades, medicalization of childbirth has led not only to a decrease in maternal and neonatal mortality but also to an increase in caesarean section rates, and in mutually unnecessary medical interventions in low risk pregnancies and births with a physiological course [4–11]. While there is no doubt about the benefit of medical interventions for mother and child in cases of abnormal processes during pregnancy and birth, medical interventions in low risk pregnancies and during physiological courses of labor may be associated with negative consequences for mother and child without a significant benefit, if used inappropriately [12–14]. New challenges include the avoidance of unnecessary medical interventions and the implementation of alternative birthing models for women who prefer little or no medical interventions [4–10].

Settings with midwife-led births are being offered as such alternatives. These maternity care and birth models are mainly realized in birth centers or at home but may also take place within the facilities of a medical institution, as a hospital-based obstetric unit. The latter provides the advantage that in case of medical complications during delivery, there is no need of transfer to a clinic, as obstetricians and the infrastructure needed for regular or emergent medical intervention (such as caesarean section or interventions in postpartum hemorrhage) are readily available. In midwife-led births, midwives are independent and self-reliant and have the sole responsibility, as long as labor and birth follows a physiological course. Otherwise, an obstetrician can be involved. Some studies suggest that midwife-led births are associated with decreased medical interventions and increased maternal satisfaction [15]. If strict selection criteria of low risk pregnant women are used, there is evidence for no increase of adverse outcomes for mother and child compared to women receiving conventional institutional settings for birth [10, 15, 16].

The University Women’s Hospital in Bern is the first university hospital in Switzerland, which has institutionalized a midwife-led birth service for women with low-risk pregnancies since 2006. When meeting the inclusion criteria, women may choose a midwife-led birth. As the published experience regarding necessity of and reasons for obstetrician involvement and its effect on maternal and neonatal outcome is limited, we aimed to address this question in our cohort of intended midwife-led births over a 14 years period of time.

## Methods

In this retrospective study we analyzed the complete consecutive cohort of all intended midwife-led births from 2006 until 2019 at the University Women’s Hospital of Bern. Women at low risk with uncomplicated pregnancies were offered a midwife-led birth. Information about our midwife-led birth model was available to women throughout the entire pregnancy (homepage, during antenatal midwife or obstetrician care, information events, pregnancy classes). Both intern and extern midwives and obstetricians could offer antenatal care to women planning a midwife-led birth. If the woman was motivated to have a midwife-led birth (free choice), she had the option to register for this midwife-led setting if well-defined criteria were met. Table 1 summarizes maternal and fetal exclusion criteria, such as complications during former pregnancies and births, maternal diseases, complications during pregnancy (placenta praevia, preeclampsia, gestational diabetes, multiple gestation, prematurity or fetal abnormalities). All women without exclusion criteria were offered a midwife-led birth when enrolling for birth at our hospital around 34 gestational weeks. Inclusion criteria were checked by either midwife or obstetrician in our outpatient clinic. The process of midwife-led birth service including possible reasons for secondary obstetrician involvement, the advantage of less medical interventions and yet the availability of any necessary intervention within the facility were discussed with all interested women. After a mandatory clinical examination and detailed ultrasonography around 34 gestational weeks, eligibility for midwife-led birth was finally decided by the head of the department. Women then were free in choosing their favored birth model. Midwife-led births took place in the same birth unit of our hospital as births with primary obstetrician-midwife team births.

**Table 1:** Maternal and fetal exclusion criteria for midwife-led birth model

anamnestic criteria	fetal criteria	maternal criteria
∙ history of caesarean section or other uterus operation ∙ history of placental retention or postpartum hemorrhage ∙ history of obstetrical anal sphincter injuries ∙ history of herpes genitalis ∙ uterus malformation ∙ in vitro fertilization/ intracytoplasmic sperm injection	∙ gestational age < 37 completed weeks ∙ multiple pregnancy ∙ oligo- /polyhydramnios ∙ fetal growth restriction ∙ fetal macrosomia ∙ congenital malformation	∙ maternal disease (e.g. diabetes, epilepsy, thrombophilia, cardiac/pulmonary disease) ∙ preeclampsia ∙ gestational diabetes ∙ placenta praevia ∙ drug abuse ∙ myoma > 5 cm ∙ vaginal bleeding ∙ active hepatitis ∙ active condylomata

Table 2 shows antepartum, intrapartum and postpartum criteria for secondary obstetrician involvement during a midwife-led birth. There could be one or multiple reasons for obstetrician involvement. Obstetrician involvement at any point during labor resulted in withdrawal from the group of midwife-led births. Inclusion and exclusion criteria for midwife-led birth as well as intrapartum and postpartum criteria for obstetrician involvement did not change during the study period of 14 years.

**Table 2:** Predefined antepartum, intrapartum and postpartum criteria for secondary obstetrician involvement

antepartum	intrapartum	postpartum
∙ vaginal bleeding ∙ non-cephalic presentation ∙ postterm gestation ≥41 completed weeks ∙ induction of labor ∙ rupture of membranes > 24 h without contractions ∙ abnormal fetal heart rate monitoring before onset of labor	∙ vaginal bleeding ∙ abnormal fetal heart rate monitoring ∙ meconium stained amniotic fluid ∙ prolonged first stage of labor ∙ prolonged second stage of labor ∙ hypertension ∙ suspicion of amniotic infection ∙ shoulder dystocia ∙ instrumental vaginal birth ∙ request of epidural anaesthesia ∙ maternal exhaustion/ decompensation	∙ postpartum hemorrhage ∙ complete or partial placental retention ∙ obstetrical anal sphincter injury ∙ severe vaginal tear

Using our prospectively collected database, we included all consecutive cases of 532 intended midwife-led births between 2006 and 2019 into the study. We excluded unplanned midwife-led births. We retrospectively analyzed the frequency of secondary obstetrician involvement and its reasons and evaluated maternal characteristics, birth mode and maternal and neonatal outcome.

This study was approved by the Ethics Committee of the Canton of Bern (Basec-No. 2016-00415).

### Statistical analysis

We used descriptive statistical analysis to examine characteristics of midwife-led births with or without secondary obstetrician involvement. We compared continuous variables by unpaired t-tests and frequency distributions of binary outcome variables by Chi-square tests using GraphPad Prism version 8.0.1. for Windows (GraphPad Software, San Diego, CA, USA) for the calculations. We considered *p*-values < 0.05 statistically significant.

## Results

**Fig. 1:**
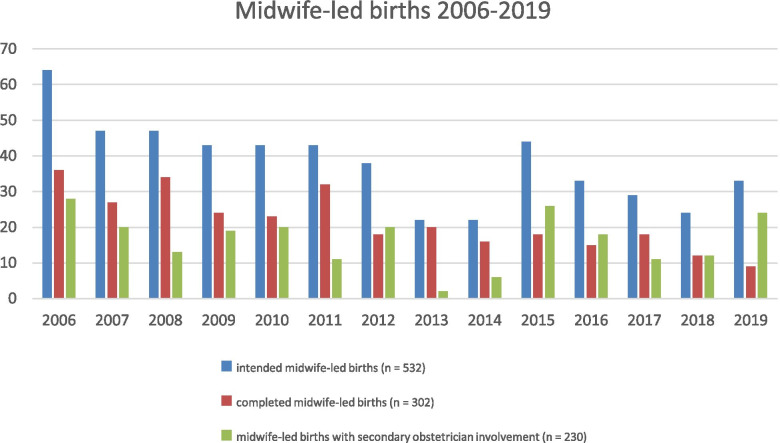
Annual numbers of intended and completed midwife-led births and midwife-led births with secondary obstetrician involvement

In our university hospital, 20,720 births took place between 2006 and 2019, among these were 532 (2.6%) women with intended midwife-led births. The annual number of births increased by about 50% during these 14 years while the proportion of intended midwife-led births declined from 5.1% in 2006 to 1.8% in 2019 (see Fig. 1).

**Fig. 2:**
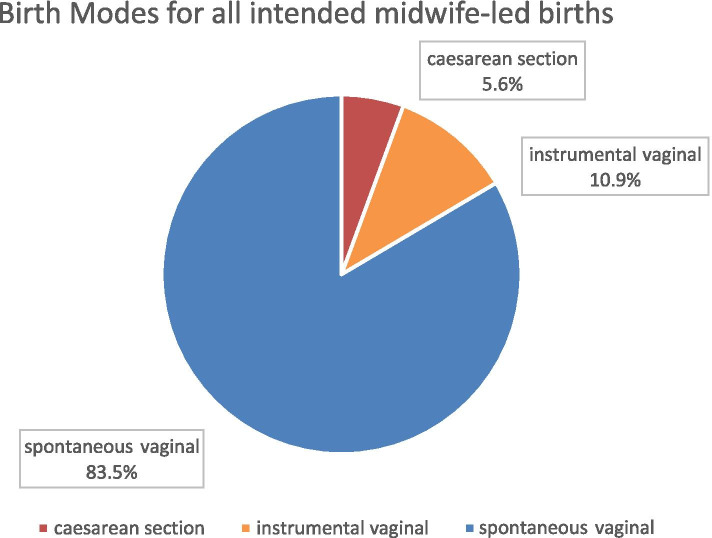
Birth Modes for all intended midwife-led births

**Fig. 3:**
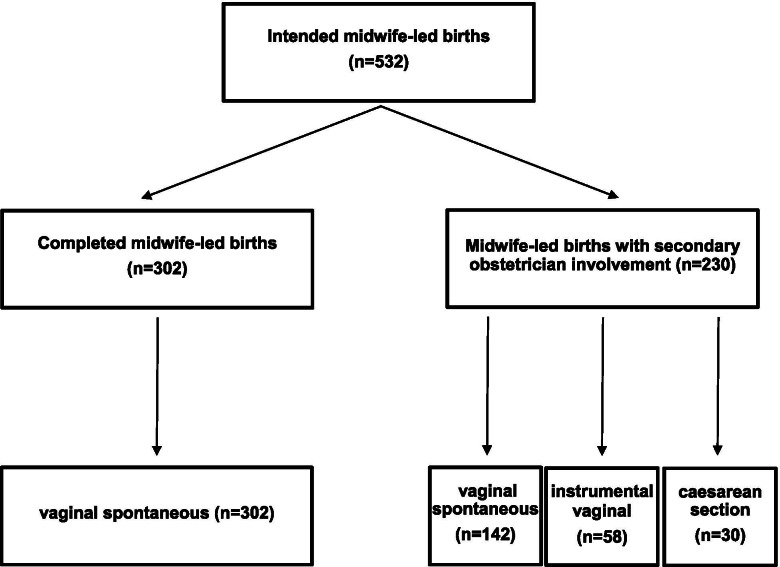
Birth Modes with and without secondary obstetrician involvement

As summarized in Fig. 2, among all intended midwife-led births, the rate of spontaneous vaginal birth was 83.5% (444/532), the rate of instrumental vaginal births was 10.9% (58/532) and the rate of caesarean section was 5.6% (30/532). 57% (302/532) women had a midwife-led birth. The remaining 230 (43%) cases required secondary obstetrician involvement (and in case of epidural analgesia also anesthesiologist involvement). Among these, 62% (142/230) had spontaneous vaginal birth, 25% (58/230) required instrumental vaginal birth and 13% (30/230) caesarean section (see Fig. 3).

Table 3 summarizes the maternal characteristics of the two groups with and without obstetrician involvement: There was no difference in the average maternal age between the two groups, but there were significantly more women ≥35 years in the group of completed midwife-led births (34% vs. 25%; *p* = 0.027). There was a significant difference in the average gestational age. More women in the group of with secondary obstetrician involvement were ≥ 41 completed gestational weeks compared to the group of completed midwife-led births (18% vs. 8%; *p* = 0.0004). There were significantly more primiparous women in the group with secondary obstetrician involvement (76% vs. 41%; *p* <  0.0001).

**Table 3:** Maternal characteristics comparing the group of completed midwife-led births to the group of midwife-led births with secondary obstetrician involvement

	completed midwife-led births (*n* = 302)	midwife-led births with secondary obstetrician involvement (*n* = 230)	*p*-value
Maternal age (years), mean ± SD	32.33 ± 4.34	31.82 ± 3.83	0.154
≥ 35 years, n (%)	103 (34.11)	58 (25.22)	*0.027*
Gestational age (weeks), mean ± SD	39.74 ± 0.93	40.06 ± 0.97	*0.0002*
≥ 41 completed gestational weeks, n (%)	24 (7.95)	42 (18.26)	*0.0004*
Primiparous women, n (%)	125 (41.39)	172 (74.78)	*< 0,0001*

*SD* Standard deviation

**Fig. 4:**
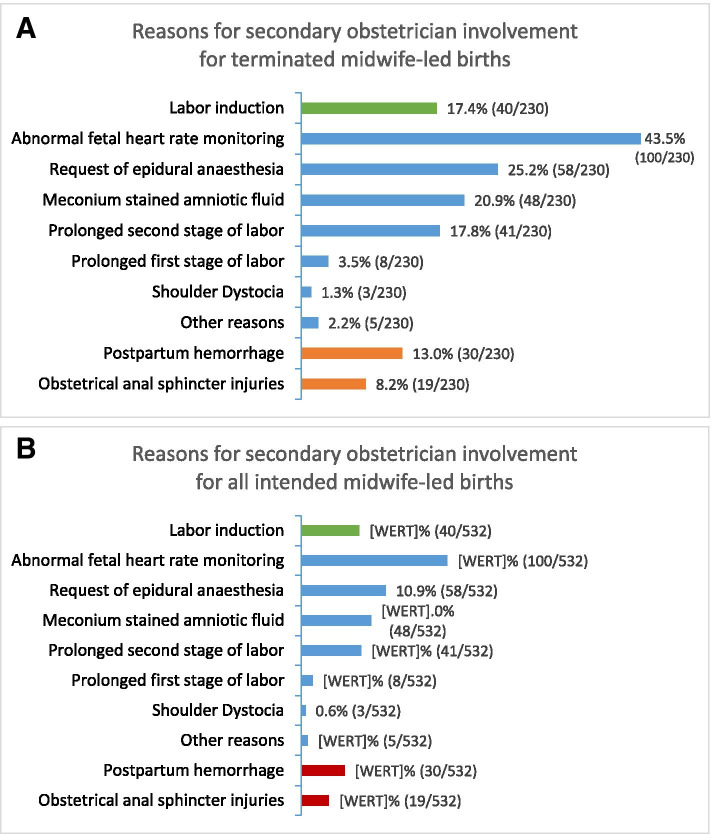
**a**: Antepartum, intrapartum and postpartum reasons for secondary obstetrician involvement in the group of terminated midwife-led births. **b**: Antepartum, intrapartum and postpartum reasons for secondary obstetrician involvement in the group of all intended midwife-led births

Figure 4 shows the possible antepartum, intrapartum or postpartum reasons for secondary obstetrician involvement for terminated midwife-led births (Fig. 4a) and all intended midwife-led births (Fig. 4b). A combination of ante-, intra- or postpartum reasons was also possible. Among 230 cases requiring obstetrician involvement, 7.4% (17/230) women had only antepartum reasons, 61.7% (142/230) women only intrapartum reasons, 13.4% (31/230) women had only postpartum reasons and 17.4% (40/230) had any combination of antepartum, intrapartum or postpartum reasons.

The results concerning the number of women requiring labor induction need to be looked at separately: need of labor induction was the most frequent antepartum reason for obstetrician involvement, primarily due to post term gestation (gestational age ≥ 41 completed weeks) or rupture of membranes without spontaneous onset of labor within 24 h after membrane rupture. Women with only antepartum reasons stated (15/17) and additionally women with a combination of antepartum, intrapartum or postpartum reasons (25/40) required labor induction, so we have a total of 40 women with labor induction among 230 midwife-led births with secondary obstetrician involvement (17.4%). Among the whole cohort of intended midwife-led births, labor induction rate was 7.5% (40/532).

Main reasons requiring secondary intrapartum obstetrician involvement included abnormal fetal heart rate monitoring in 43.5% (100/230), epidural analgesia in 25.2% (58/230), meconium stained amniotic fluid in 20.9% (48/230), prolonged second stage of labor in 17.8% (41/230) or first stage of labor in 3.5% (8/230). Indications for caesarean sections were mainly abnormal fetal heart rate monitoring, prolonged first or second stage of labor and fetal malposition.

Among postpartum complications we found postpartum hemorrhage (with or without placental retention) in 13% (30/230) of births with secondary obstetrician involvement. The overall rate of postpartum hemorrhage was 5.6% (30/532). 5.2% (12/230) women required postpartum secondary obstetrician involvement solely due to obstetrical anal sphincter injuries, while 3.0% (7/230) obstetrical anal sphincter injuries occurred after obstetrician involvement due to ante- and intrapartum complications. The overall rate of obstetrical anal sphincter injuries in the whole cohort was 3.6% (19/532). A comparison of the incidence of postpartum hemorrhage and obstetrical anal sphincter injuries between the two groups with and without obstetrician involvement is not possible, as both events were considered as postpartal complications leading to termination of midwife-led birth.

Regarding maternal outcome, the episiotomy rate was significantly higher in the group with secondary obstetrician involvement (30.9% versus 3.6%; *p* <  0.0001) (see Table 4). The overall episiotomy rate in the whole cohort was 15.4%. Indication for episiotomy in the group with secondary obstetrician involvement was mainly abnormal fetal heart rate monitoring (66.2%) in spontaneous vaginal births or instrumental vaginal births.

**Table 4:** Maternal and neonatal outcome comparing the group of completed midwife-led births to the group of midwife-led births with secondary obstetrician involvement

	completed midwife-led births (*n* = 302)	midwife-led births with secondary obstetrician involvement (*n* = 230)	*p*-value
Episiotomy, n (%)	11 (3.64)	71 (30.87)	*< 0,0001*
Birth weight (g), mean ± SD	3363 ± 345	3435 ± 407	*0.028*
5′ Apgar < 7, n (%)	0 (0)	2 (0,87)	0,105
Arterial umbilical cord pH < 7.10 (all birth modes), n (%)	4 (1.70)	13 (6.25)	*0.013*
NICU, n (%)	1 (0.33)	6 (2.61)	*0.022*

*SD* Standard deviation, *NICU* Neonatal intensive care unit

Table 4 shows the results concerning neonatal outcome. There was a significant difference in the birth weights of the newborns: Newborns in the group with secondary obstetrician involvement weighed more than newborns in the group of completed midwife-led births (*p* = 0.028). There was no difference regarding the incidence of Apgar scores at 5′ < 7 for the two groups of midwife-led births with and without secondary obstetrician involvement (*p* = 0.105). Between the two groups there was a significant difference in the rate of arterial umbilical cord pH < 7.10 when including all birth modes (*p* = 0.013) and when comparing the subgroup of spontaneous vaginal births (*p* = 0.004). The overall rate of arterial umbilical cord pH < 7.10 was 3.2%. The transfer rate of newborns to the neonatal intensive care unit (NICU) was 1.3% for all intended midwife-led births. In the group of completed midwife-led births, 1 newborn was transferred because of acute respiratory syndrome, whereas in the group with secondary obstetrician involvement 6 newborns required transfer to the NICU due to maladaptation, acute respiratory syndrome, possible meconium aspiration and infection/−sepsis (*p* = 0.022).

## Discussion

Our study summarizes 14 years of experience of midwife-led births in an obstetric department of a university hospital. Main findings of our study are the low overall medical intervention rate, the good maternal and neonatal outcome and the significant proportion of secondary obstetrician involvement before, during or after delivery.

After all, 43% of women with an intended midwife-led birth had ante-, intra- or postpartum obstetrician involvement at some point. This number seems rather high, in view of the fact that women planning a midwife-led birth were already pre-selected according to their medical history and course of pregnancy. As can be expected, obstetrician involvement was highest in primiparous women. Women at ≥41 completed gestational weeks required labor induction, this explains the higher percentage of women with advanced gestational age in the group of women with secondary obstetrician involvement.

Nevertheless, the proportion of vaginal births (spontaneous or instrumental) was high and the overall caesarean section rate was low. Also the rate of other medical interventions (e.g. induction of labor, episiotomy) was low. Obstetrical anal sphincter injuries and postpartum hemorrhage with or without placental retention were as expected, comparable to the total population in the department. Our current policy is offering a hospital-based midwife-led birth model to low risk women. The results of our study support the appropriateness of the predefined exclusion criteria as well as the predefined criteria for secondary obstetrician involvement for our midwife-led birth model.

Overall, maternal and neonatal outcome in the complete cohort of all intended midwife-led births was very good and the overall transfer rate of newborns to the NICU was low. The higher rate of arterial umbilical cord pH < 7.10 and transfer of newborns to the NICU in the group of midwife-led births with secondary obstetrician involvement reflects ante-, intra- and postpartum complications and indirectly supports the appropriateness of our criteria to involve an obstetrician for the safety of mother and child, when these criteria are met.

Regarding the frequency of obstetrician involvement, Bodner-Adler et al. describe a low transfer rate in their study of midwife-led care at a tertiary care center in Austria [17]: In contrast to our results, the secondary obstetrician involvement among midwife-led births in low risk women in the study by Bodner-Adler et al. was only 7% of a total of 2123 intended midwife-led births over a period of 10 years. One important explanation for the discrepancy to our results may be that the majority of women (74%) in their study were multiparous women. Another explanation might be a different definition of obstetrician involvement. The caesarean section rate among these births was 7%, while 93% women had spontaneous or instrumental vaginal births. Matched with low risk women assigned to primarily obstetrician-led births there was a significant decrease in interventions and no adverse maternal or neonatal outcomes [17].

Another more recent study of midwife-led care during birth at a tertiary care center in Germany by Merz et.al. showed a similar obstetrician involvement rate as our study (50%), while the caesarean section rate (9.3%) in the intended midwife-led birth group was almost the double of the rate in our study. The authors found higher odds for transfer from midwife-led births to standard obstetric care for nulliparous women, higher age and increased birthweight (+ 100 g) [18].

It needs to be noted that the rate of 2.6% of intended midwife-led births compared to the total number of births at our institution was low. This may have several reasons: One reason might be that the proportion of high risk pregnancies at our tertiary care perinatal center is high and thus many women not meeting our relatively strict selection criteria are excluded. Additionally, during the evaluated 14 years, the number of births accompagnied by free practicing midwife in our hospital increased, which in fact may be a direct competition to our midwife-led delivery model with resident midwives. Furthermore, our midwife-led birth setting within the hospital might not be widespread known or the hospital-based setting and atmosphere does not meet some womens’ expectations of a natural birth. Results of our study including favorable maternal and neonatal outcome and low medical intervention rate in a hospital-based but midwife-led birth setting can be used to better inform women in order to make a well-informed choice and help making our in-hospital midwife-led birth model more popular.

Maillefer et al. found that women and health professionals are favourable towards the development of midwife-led units in university hospitals, women notably focusing on the continuity of care [19]. Many women with low risk pregnancies wish to give birth in the most natural way as possible, with as little medical interventions as necessary and with continuity of care in a non-medical atmosphere, involving as little attending people as possible. They want to ensure safety for themselves and their child. Systematic reviews comparing midwife-led continuity models to other models of care for childbearing women provide good evidence that low risk pregnant women under midwife care experience less medical intervention and more satisfaction with at least comparable adverse outcomes [15, 16]. Our results and experience with a low overall medical intervention rate and favorable maternal and neonatal outcome are in line with evidence-based benefits of midwife-led maternal care and birth models.

We need to emphasize on the advantages of an in-hospital midwife-led birth model, where the same rooms are being used and secondary obstetrician consultation is available immediately and at any time. As medical interventions in low risk pregnancies and during physiological course of labor are rather associated with negative consequences than significant benefits for mother and child [12–14], more interest should focus on avoidance of unnecessary interventions, continuous consideration of interventions’ appropriateness and well defined selection criteria for midwife-led maternity care and birth models.

Our study concentrated on women with low risk pregnancies. Current studies are now focusing on the evaluation of clinical- and cost-effectiveness in midwifery care models for women experiencing complex pregnancy and women with chronic medical conditions [20–22]. In a randomized controlled trial comparing midwife-coordinated maternity care intervention with standard care for women with chronic medical conditions, de Wolff et al. found an increased level of satisfaction with maternity care among women who received midwife coordinated maternity care intervention [21].

A further important aspect is the view of midwives in our team. The option of keeping full responsibility for a woman during childbirth in a midwife-led birth setting is sometimes challenging, but at the same time very satisfying. Interestingly the authors believe that even the team spirit between midwives and obstetricians is promoted by midwife-led births.

In summary, midwife-led birth settings within a clinical obstetric department offer primary and continuous care by a midwife without missing out on the advantages of the hospital’s facilities and infrastructure, ensuring safety for mother and child during the entire course of labor. According to our study results and our daily experience, an in-hospital midwife-led birth model for women with low risk pregnancies is safe and represents an interesting offer to women looking for less “medicalized” birth care. Nevertheless, it must be considered that in almost half of the cases an obstetrician involvement is necessary. Despite the high rate of obstetrician involvement, the caesarean section rate as well as the instrumental vaginal birth rate and episiotomy rate are very low and maternal and neonatal outcome is good. This is probably achieved by using the same hospital’s facilities and resources for midwife-led births as well as for standard midwife-obstetrician-led births in case of complications at any time. A further positive effect is that the midwives and doctors work together in the same unit as a team and are not artificially separated.

The retrospective study design, the small numbers and the lack of matching with primarily obstetrician-led births limits the generalizability of our findings and applicability in practice. Data about medical intervention rate and maternal and neonatal outcome of low risk women with obstetrician-led births were not evaluated. Our results only focus on midwife-led births in low risk pregnancies including those with secondary obstetrician involvement. Extrapolation of outcomes for the entire collective of low risk women therefore is not possible and limits a general statement. Future evaluations should focus on the criteria and appropriateness of specialist’s referral used to determine antepartum care and birth models offered to women.

While systematic reviews of midwifery care, mostly conducted in the United Kingdom, provide good evidence for cost effectiveness in midwife-led birth models for women with low risk pregnancies, information on cost effectiveness of midwifery care for women with complex pregnancy is limited [15, 16, 20, 23, 24]. As cost effectiveness needs to interpreted in relation to the different health systems in Europe and to our knowledge there is no such evaluation for Switzerland, an economic evaluation of our midwife-led birth model compared to the standard care within our setting is required.

## Conclusion

Midwife-led birth care for women with low risk pregnancies was demonstrated to be a safe alternative to midwife-obstetrician-led birth care within the setting of a university hospital with a maternity birthing service. Prerequisites for the success of a midwife-led birth model of care include rigorous evidence-based assessment tools to identify low risk women, clear criteria for obstetrician involvement (and if needed, anesthesiologist and neonatologist consultation) and a clearly structured organization within an obstetric facility to offer women choice. These study findings contribute to the growing evidence-base that supports midwifery continuity of care models that facilitate choice for women and safe outcomes for mothers and their babies.

## Data Availability

The datasets used and analysed during the current study are available from the corresponding author on request.

## Abbreviations

NICU: Neonatal intensive care unit
SD: Standard deviation

## References

[1] Bundesamt für Statistik. Geburten-Todesfälle, Geburten. https://www.bfs.admin.ch/bfs/de/home/statistiken/bevoelkerung/geburten-todesfaelle/geburten.html. Accessed 2020.

[2] Bundesamt für Statistik. Gesundheitsstatistik 2019. https://www.bfs.admin.ch/bfs/de/home/statistiken/gesundheit.assetdetail.10227275.html. Accessed 29 Oct 2019.

[3] Schweizerischer Hebammenverband. Statistikbericht der frei praktizierenden Hebammen. https://www.hebamme.ch/qualitaet/statistikberichte-fph/. Accessed Sept 2020.

[4] Euro-Peristat project in collaboration SCPE, EUROCAT and EURONEOSTAT. European Perinatal Health Report 2004. https://www.europeristat.com/index.php/reports/european-perinatal-health-report2004.html. Accessed 11 Dec 2008.

[5] Zeitlin J, Mohangoo A, Cuttini M, Alexander S, Barros H, EUROPERISTAT report writing committee (2009). The European perinatal health report: comparing the health and care of pregnant women and newborn babies in Europe. J Epidemiol Community Health.

[6] Euro-Peristat project with SCPE and Eurocat. European Perinatal Health Report. The health and care of pregnant women and babies in Europe in 2010. https://www.europeristat.com/index.php/reports/european-perinatal-health-report-2010.html. Accessed 27 May 2013.

[7] Zeitlin J, Mohangoo AD, Delnord M, Cuttini M, Euro-Peristat scientific committee (2013). The second European perinatal health report: documenting changes over 6 years in the health of mothers and babies in Europe. J Epidemiol Community Health.

[8] Euro-Peristat Project. European Perinatal Health Report. Core indicators of the health and care of pregnant women and babies in Europe in 2015. https://www.europeristat.com/index.php/reports/european-perinatal-health-report-2015.html. 26 Nov 2018.

[9] Zeitlin J, Alexander S, Barros H, Blondel B, Delnord M, Gissler M, For the euro-Peristat scientific committee (2019). Perinatal health monitoring through a European lens: eight lessons from the euro-Peristat report on 2015 births. BJOG..

[10] Hodnett ED, Downe S, Walsh D, Cochrane pregnancy and childbirth group (2012). Alternative versus conventional institutional settings for birth. Cochrane Database Syst Rev.

[11] Eide BI, Nilsen ABV, Rasmussen S (2009). Births in two different delivery units in the same clinic – A prospective study of healthy primiparous women. BMC Pregnancy Childbirth.

[12] Villar J, Carolli G, Zavaleta N, Donner A, Wojdyla D, Faundes A (2007). Maternal and neonatal individual risks and benefits associated with caesarean delivery: a multicentre prospective study. BMJ..

[13] Macfarlane AJ, Blondel B, Mohangoo AD, Cuttini M, Nijhuis J, Novak Z (2016). Wide differences in mode of delivery within Europe: risk-stratified analyses of aggregated routine data from the euro-Peristat study. BJOG..

[14] Davis-Floyd R, Barclay L, Tritten J (2009). Birth models that work.

[15] Sandall S, Soltani H, Gates S, Shennan A, Devane D (2016). Midwife-led continuity models versus other models of care for childbearing women. Cochrane Database Syst Rev.

[16] Sutcliffe K, Caird J, Kavanagh J, Rees R, Oliver K, Dickson K (2012). Comparing midwife-led and doctor-led maternity care: a systematic review of reviews. J Adv Nurs.

[17] Bodner-Adler B, Kimberger O, Griebaum J, Husslein P, Bodner K (2017). A ten-year study of midwife-led care at an Austrian tertiary care center: a retrospective analysis with special consideration of perineal trauma. BMC Pregnancy Childbirth.

[18] Merz WM, Tascon-Padron L, Puth M-T, Heep A, Tietjen SL, Schmid M (2020). Maternal and neonatal outcome of births planned in alongside midwifery units: a cohort study from a tertiary center in Germany. BMC Pregnancy Childbirth.

[19] Maillefer F, de Labrusse C, Cardia-Vonèche L, Hohlfeld P, Stoll B (2015). Women and healthcare providers’ perceptions of a midwife-led unit in a Swiss university hospital: a qualitative study. BMC Pregnancy Childbirth.

[20] Donnellan-Fernandez RE, Creedy DK, Callander EJ (2018). Cost-effectiveness of continuity of midwifery care for women with complex pregnancy: a structured review of the literature. Health Econ Rev.

[21] de Wolff MG, Midtgaard J, Johansen M, Rom AL, Rosthøj S, Tabor A (2021). Effects of a Midwife-Coordinated Maternity Care Intervention (ChroPreg) vs. Standard Care in Pregnant Women with Chronic Medical Conditions: Results from a Randomized Controlled Trial. Int J Environ Res Public Health.

[22] Fernandez Turienzo C, Bick D, Briley AL, Bollard M, Coxon K, Cross P, et al. Midwifery continuity of care versus standard maternity care for women at increased risk of preterm birth: A hybrid implementation-effectiveness, randomised controlled pilot trial in the UK. PLoS Med. 2020;17(10):e1003350. 10.1371/journal.pmed.1003350 PMC7537886 33022010

[23] Devane D, Brennan M, Begley C, Clarke M, Walsh D, Sandall J (2010). Socioeconomic value of the midwife: a systematic review, meta-analysis, meta-synthesis and economic analysis of midwife-led models of care.

[24] Ryan P, Revill P, Devane D, Normand C (2013). An assessment of the cost-effectiveness of midwife-led care in the United Kingdom. Midwifery.

